# Metabolic syndrome and poor self-rated health as risk factors for premature employment exit: a longitudinal study among 55 016 middle-aged and older workers from the Lifelines Cohort Study and Biobank

**DOI:** 10.1093/eurpub/ckad219

**Published:** 2023-12-18

**Authors:** Katharina Runge, Sander K R van Zon, Kène Henkens, Ute Bültmann

**Affiliations:** Work and retirement theme group, Netherlands Interdisciplinary Demographic Institute, The Hague, The Netherlands; Department of Health Sciences, Community and Occupational Medicine, University of Groningen, University Medical Center Groningen, Groningen, The Netherlands; Department of Health Sciences, Community and Occupational Medicine, University of Groningen, University Medical Center Groningen, Groningen, The Netherlands; Work and retirement theme group, Netherlands Interdisciplinary Demographic Institute, The Hague, The Netherlands; Department of Health Sciences, Community and Occupational Medicine, University of Groningen, University Medical Center Groningen, Groningen, The Netherlands; Faculty of Social and Behavioral Sciences, University of Amsterdam, Amsterdam, The Netherlands; Department of Health Sciences, Community and Occupational Medicine, University of Groningen, University Medical Center Groningen, Groningen, The Netherlands

## Abstract

**Background:**

Poor self-rated health (SRH) is a well-established risk factor for premature employment exit through unemployment, work disability, and early retirement. However, it is unclear whether the premature employment exit risk associated with underlying cardio-metabolic health conditions is fully captured by poor SRH. This study examines the metabolic syndrome (MetS), an early-stage risk factor for cardiovascular disease and type two diabetes mellitus, as a risk factor for premature employment exit while controlling for poor SRH.

**Methods:**

We analyzed data from *N* = 55 016 Dutch workers (40–64 years) from five waves of the Lifelines Cohort Study and Biobank. MetS components were based on physical measures, blood markers, and medication use. SRH and employment states were self-reported. The associations between MetS, SRH, and premature employment exit types were analyzed using competing risk regression analysis.

**Results:**

During 4.3 years of follow-up, MetS remained an independent risk factor for unemployment [adjusted subdistribution hazard ratio (SHR): 1.14, 95% CI: 1.03, 1.25] and work disability (adjusted SHR: 1.33, 95% CI: 1.11, 1.58) when adjusted for poor SRH, common chronic diseases related to labor market participation (i.e., cancer, musculoskeletal-, pulmonary-, and psychiatric diseases), and sociodemographic factors. MetS was not associated with early retirement.

**Conclusions:**

Poor SRH did not fully capture the risk for unemployment and work disability associated with MetS. More awareness about MetS as a ‘hidden’ cardio-metabolic risk factor for premature employment exit is needed among workers, employers, and occupational health professionals. Regular health check-ups including MetS assessment and MetS prevention might help to prolong healthy working lives.

## Introduction

Despite increasing efforts to extend working lives in Western countries, many middle-aged and older workers prematurely exit employment, i.e., they stop working before reaching the statutory retirement age.^1^ Existing literature consistently showed that poor self-rated health (SRH) is a major risk factor for premature employment exit especially through work disability and unemployment, and to a lesser degree through early retirement.^2–4^ SRH is easily assessed by a single self-reported question and has been identified as a strong risk factor for mortality.^5^ Further, SRH is widely assumed to be an accurate reflection of participant’s evaluation of their overall health status, e.g., in terms of presence or absence of disease or functional limitations.^6^ To achieve a precise estimation of health-related premature employment exit risks, SRH measures rely on the awareness of workers about having specific underlying health conditions. Earlier research demonstrated, however, that individuals are often not aware of early-stage physiological processes, such as critical biomarker levels, which can reflect pre-disease pathways and may increase the risk for premature employment exit.^7^

Numerous studies have investigated the effect of SRH on premature employment exit along with more elaborate self-reported health measures, such as chronic diseases and diagnosed health conditions,^2^^,^^8–12^ or body mass index (BMI).^13^^,^^14^ Further, scholars have started to jointly investigate the effect of SRH and externally assessed health indicators, such as grip strength or obesity, e.g., by a medical professional, on premature employment exit.^15^^,^^16^ These external assessed health indicators might point to ‘hidden’ health risks for premature labor market exit. For instance, Kalwij and Vermeulen^15^ showed the added value of including on-site assessed grip strength in addition to SRH in models to predict labor market participation. Further, Månsson and Merlo^16^ identified poor SRH and on-site assessed obesity (i.e. a BMI of ≥30) as independent risk factors for work disability. This study will expand this line of research by testing whether the metabolic syndrome (MetS), an early-stage (i.e., pre-disease) cardio-metabolic risk factor, constitutes a ‘hidden’ risk factor for premature employment exit, which is not fully captured in SRH ratings.

Recently, MetS has been identified as a modifiable and early-stage risk factor for premature employment exit through work disability among middle-aged and older workers.^17^ MetS is a cardio-metabolic health measure, which consists of a combination of at least three out of the following five components: hypertension, abdominal obesity, raised fasting plasma glucose, raised triglycerides, and lowered high-density lipoprotein cholesterol.^18^ MetS has been declared a global epidemic due to increasing MetS prevalence and incidence rates worldwide.^19^ The total global MetS prevalence is estimated to be 25%, i.e., one out of four people classify for MetS.^19^ MetS development is closely related to an unhealthy lifestyle consisting of modifiable health behaviors, such as physical inactivity, an unhealthy diet, smoking, and/or high alcohol consumption.^20^ Further, MetS is an early-stage risk factor for the development of chronic diseases, such as cardiovascular disease (CVD) and type two diabetes mellitus (T2DM),^18^ which both increase the risk for premature employment exit.^21^^,^^22^

The aim of this study is to examine whether MetS remains a risk factor for premature employment exit while controlling for poor SRH and common chronic diseases related to labor market participation. If MetS additionally adds to the premature employment exit risk estimate, this would indicate that SRH measures do not fully capture the ‘hidden’ premature labor market exit risk associated with MetS. This information could be relevant for preventive measures aimed at prolonging healthy working lives.

## Methods

### Study design and sample

The present longitudinal study was embedded within the large-scale Lifelines Cohort Study and Biobank.^23^ Lifelines is a multi-disciplinary, prospective population-based cohort study examining the health and health behaviors of 167 729 persons (baseline age: 0–93 years) living in the North of the Netherlands. The Lifelines population is broadly representative of the population of the North of the Netherlands on sociodemographic characteristics, lifestyle factors, chronic disease prevalence, and general health.^24^ Lifelines employs a broad range of investigative procedures in assessing the biomedical, sociodemographic, behavioral, physical, and psychological factors, which contribute to the health and disease of the general population. Eligible participants and their family members were recruited through general practitioners and online self-registration. The ongoing data collection started in 2007 with a comprehensive baseline assessment (T0) at a Lifelines research center, including filling out extensive questionnaires, collecting biological samples, and a physical examination.^23^ On average, every 1.5 years, follow-up questionnaires are completed (T1, T2 and T4). Approximately 5 years after T0, participants re-visited a Lifelines research center for the second comprehensive assessment (T3).^23^ This study includes information from the first five measurement waves (T0–T4) with a total median follow-up time of 4.3 years (SD: 1.9 years). The data collection of the Lifelines Cohort Study and Biobank was conducted according to the principles of the Declaration of Helsinki and was approved by the medical ethics committee of the University Medical Center Groningen (2007/152).

Participants were included in this study sample if they were middle-aged and older workers at T0 (i.e., 40–64 years old and working ≥12 h per week, which is in line with the Dutch definition of employment at the time of the data collection^25^), do not self-report to have CVD and/or T2DM at T0 (for more details on the coding of CVD and T2DM see Reference^26^), and have information on at least one follow-up employment status measure after T0. Consequently, from the Lifelines adult study population at T0 (aged 18–93 years, *N* = 152 728), participants were excluded who did not meet the age criterion (42.6%, *N* = 65 106), worked <12 h per week at T0 (13.2%, *N* = 20 159), reported to have CVD and/or T2DM (4.7%, *N* = 7191), were lost to follow-up after T0 (3%, *N* = 4587), and did not have any follow-up measure of employment status after T0 (0.4%, *N* = 595). Lastly, we excluded participants in the International Standard Classification of Occupations (ISCO) 08 category ‘armed forces’ (0.05%, *N* = 74) due to the heterogeneity within this group as no task is specified for this ISCO-08 category and because of the small sample size.^27^ The final study sample included *N* = 55 016 participants.

### Measures

#### Premature employment exit

Employment status was measured at every assessment wave by the question ‘Which situation applies to you?’ including the following answer options: working for pay (including the number of hours per week), unemployed (registered with the job center), work disabled, homemaker, student, retiree (age 65), early retiree, or other. Participants could select several answer options and paid work was given priority in the coding of premature employment exit over the other response options. Based on the first reported employment status change after T0, the following premature employment exit types were compiled: unemployment, work disability, and early retirement. The group ‘working at the end of follow-up’ consists of participants who reported to be continuously in employment during follow-up. Participants who reached the state pension age of 65 or became economically inactive (i.e., employment states homemaker, student or other) were censored. Participants with missing values on employment status during follow-up were included if at least one follow-up employment status after T0 was reported.

#### Self-rated health

SRH was assessed at T0 by the following question of the RAND 36-item Health Survey: ‘In general, would you say your health is …?’ with response options on a 5-point scale.^28^ Responses were coded into good health (excellent, very good, and good) and poor health (fair and poor).

#### Metabolic syndrome

MetS was assessed at T0 using the joint interim criteria.^18^ For a MetS classification, at least three out of five components need to be present: abdominal obesity [waist circumference (WC) ≥88 cm in females, WC ≥ 102 cm in males], raised triglycerides (≥1.7 mmol/l) or medication to treat lipid abnormalities [Anatomical Therapeutic Chemical (ATC) code C10A or C10B],^29^ lowered HDL-Cholesterol (<1.0 mmol/l in males, <1.3 mmol/l in females) or medication to treat lipid abnormalities (ATC code C10A or C10B),^29^ hypertension (systolic ≥130 and/or diastolic ≥85 mm Hg) or hypertension treatment (ATC code C02, C03, C07, C08 or C09),^29^ raised blood glucose (fasting ≥5.6 mmol/l, non-fasting ≥11 mmol/l) or medication for T2DM (ATC codes A10A or A10B).^18^^,^^29^ MetS components were assessed by trained research staff using standardized protocols and calibrated measurement instruments.^23^ WC was measured in an upright position and in the middle between the front end of the lower ribs and the iliac crest. Triglycerides, HDL-cholesterol, and fasting glucose were assessed by fasting blood samples. Systolic and diastolic blood pressure was based on the mean value of the final three measurements from an automatic blood pressure monitor.^30^ MetS was included as a binary variable (yes/no) in the main analyses.

### Covariates

#### Chronic diseases

Common chronic diseases related to labor market participation (i.e., cancer, musculoskeletal-, pulmonary-, and psychiatric diseases) were included as separate binary variables (yes/no) in the analyses.^31^ Musculoskeletal diseases (arthrosis, osteoporosis, and/or rheumatoid arthritis), pulmonary diseases (asthma and/or chronic obstructive pulmonary disease), cancer and psychiatric diseases (depression and/or anxiety disorder) were assessed by self-report at T0 with the question ‘Have you (had) one of the following conditions?’.^26^

#### Sociodemographic factors

Age, sex, partner status, educational level, weekly working hours, and occupational group membership were self-reported at T0. ‘Partner status’ was categorized as married/partnered and not married/partnered. ‘Educational level’ was categorized as low (no education; primary education; lower or preparatory secondary vocational education; junior general secondary education), medium (secondary vocational education or work-based learning; senior general secondary education, pre-university secondary education), or high (higher vocational education; university education).^32^ ‘Weekly working hours’ were measured on a continuous scale ranging from 12 to 40 h per week. ‘Occupational group membership’ was coded according to the ISCO-08 by Statistics Netherlands.^27^^,^^33^ Based on the major ISCO-08 categories, four overarching occupational groups were compiled: high-skilled white-collar (i.e. managers, professionals, technicians and associate professionals), low-skilled white-collar (i.e. clerical support workers, services and sales workers), high-skilled blue-collar (i.e. skilled agricultural forestry and fishery workers, craft and related trades workers), and low-skilled blue-collar workers (i.e. plant and machine operators and assemblers and elementary occupations).^34^

### Multiple imputation

Multiple imputation was used to handle missing data on occupational group membership (2.7%, *N* = 1471), weekly working hours (0.9%, *N* = 514), MetS components (highest missing 0.9%, *N* = 488), SRH (0.3%, *N* = 142), educational level (0.1%, *N* = 36), and partner status (0.0%, *N* = 25). Imputations were predicted by follow-up time, age, sex, chronic diseases, and follow-up employment status. In total, ten datasets were imputed.^35^

### Statistical analyses

Baseline MetS, MetS components, SRH, chronic diseases, and sociodemographic characteristics were investigated for the total study sample, separately for participants who still worked at the end of follow-up, and by premature employment exit type. Further, descriptive baseline characteristics were compared between the study sample and participants who were lost to follow-up or did not have any follow-up information on employment status. Lastly, the individual and joint effects of MetS and SRH on premature employment exit were examined by means of Fine & Gray’s proportional subdistribution hazards survival analysis.^36^ This analysis takes into account that one employment exit type ‘prevents’ the occurrence of other employment exit types by controlling for competing exit routes from employment.^37^ Subdistribution hazard ratios (SHRs) and corresponding 95% confidence intervals (95% CIs) were listed for MetS, SRH, chronic diseases, and sociodemographic factors. An individual time-to-event variable is by default included in the analysis to take differing follow-up times of participants into account. In Models 1 and 2, the individual effects of MetS and poor SRH on premature employment exit types were examined. In Model 3, the joint effect of MetS and poor SRH on premature employment exit types was investigated to examine whether the effect of MetS on premature employment exit can be fully captured by poor SRH. All models were adjusted for sociodemographic factors and chronic diseases.^31^ Data preparation, multiple imputation of missing values, and descriptive analyses were conducted using IBM SPSS Statistics version 28. Competing risk regression analyses were carried out in StataMP 13.1.

### Sensitivity analyses

The robustness of the results was tested by repeating the analyses among all working participants, i.e., also including participants who work 1 h or more but <12 h per week at baseline. This working hour selection is in line with the current international definition of employment.^38^ Further, all analyses were repeated using different codings of the main risk factors MetS (i.e. a categorical variable consisting of the number of MetS components ranging from 0 to 5 and using three MetS components as the reference category—to investigate whether a dose–response relationship exists between an increasing number of MetS components and premature employment exit types) and SRH (i.e. a continuous variable ranging from 1 to 5). Lastly, the individual MetS components were investigated as risk factors for premature employment exit in separate analyses.

## Results

### Sample characteristics

The baseline mean age of the study sample (*N* = 55 016) was 48.1 years (SD: 5.7) (table 1). At baseline, 15.2% of participants classified for MetS (*N* = 8351) and 6.5% reported poor SRH (*N* = 3566). The baseline prevalence of chronic diseases was highest among participants who became work disabled during follow-up, with the highest reported prevalence of psychiatric diseases (25.9%, *N* = 189). After a median follow-up time of 4.3 years (SD: 1.9 years), 5.5% (*N* = 3036) participants became unemployed (mean time to event: 3.0 years, SD: 1.8 years), 1.3% (*N* = 729) work disabled (mean time to event: 3.0 years, SD: 1.9 years), and 1.9% (*N* = 1060) retired early (mean time to event: 3.2 years, SD: 1.8 years). Further, 4.8% of participants were censored because of reaching the age of 65 years (*N* = 1921) or becoming economically inactive (total *N* = 707; consisting of *N* = 495 homemakers, *N* = 35 students, and *N* = 177 employment status ‘other’). Compared to the study sample, those participants who were lost to follow-up after baseline (*N* = 4587) or did not have any follow-up measure on employment status after baseline (*N* = 595) had a higher prevalence of MetS and poor SRH at baseline (Supplementary table S1).

**Table 1:** Baseline (T0) characteristics of the total study sample, working at the end of follow-up, and premature employment exit types

			Premature employment exit		
	Total study sample	Working at the end of follow-up	Unemployment	Work disability	Early retirement
	*N* = 55 016	*N* = 47 563	*N* = 3036	*N* = 729	*N* = 1060
	% or mean (SD)	% or mean (SD)	% or mean (SD)	% or mean (SD)	% or mean (SD)
Health status					
MetS	15.2	14.6	17.8	23.6	21.4
MetS components					
Abdominal obesity	35.1	34.4	38.5	47.9	38.2
Hypertension	42.8	41.7	45.4	49.7	54.4
Raised triglycerides	18.9	18.6	20.1	25.4	23.7
Reduced HDL-cholesterol	14.6	14.3	16.7	22.9	17.5
Raised blood glucose	12.3	11.6	14.4	16.9	19.5
Poor self-rated health	6.5	6.0	8.3	33.2	5.5
Chronic diseases					
Musculoskeletal	2.7	2.4	3.0	7.0	5.6
Pulmonary	10.4	10.3	10.9	16.2	8.3
Cancer	4.2	3.8	4.8	7.7	8.8
Psychiatric	11.3	10.7	15.7	25.9	11.7
Sociodemographic factors					
Age (years)	48.1 (5.7)	47.2 (4.9)	48.7 (5.8)	49.4 (5.9)	59.1 (2.7)
Male sex	45.8	46.1	42.7	40.7	50.9
Not married/partnered	10.0	9.5	15.5	15.1	6.8
Occupational group					
High-skilled white-collar	50.0	50.9	42.3	35.7	59.9
Low-skilled white-collar	31.0	30.4	37.8	32.9	27.3
High-skilled blue-collar	10.7	10.8	9.7	15.2	7.7
Low-skilled blue-collar	8.2	7.9	10.2	16.2	5.1
Educational level					
High	31.0	31.6	23.7	18.9	37.5
Medium	40.0	41.1	38.8	36.4	27.4
Low	27.4	25.7	35.8	43.1	33.5
Other	1.7	1.6	1.7	1.6	1.6
Weekly working hours	31.4 (8.7)	31.7 (8.6)	30.7 (8.8)	28.2 (9.8)	29.4 (8.7)

Note: SD, standard deviation; MetS, metabolic syndrome; HDL, high-density lipoprotein.

### Baseline MetS and SRH status and the risk of premature employment exit during follow-up

In table 1, the baseline prevalence of MetS and poor SRH is presented by 4.3-year follow-up employment states. The highest MetS prevalence was measured among participants who prematurely exited employment during follow-up through work disability (23.6%), followed by early retirement (21.4%), unemployment (17.8%), and lastly participants who still worked at the end of follow-up (14.6%). Poor SRH at baseline was most common among participants who became work disabled during follow-up (33.2%), followed by unemployment (8.3%), participants who still worked at the end of follow-up (6.0%), and early retirees (5.5%). The highest baseline prevalence of hypertension (54.4%) was assessed among workers who became early retirees during follow-up while the highest prevalence of abdominal obesity (47.9%) was present among workers who became work disabled.

In tables 2–4, the prospective associations between MetS, SRH, and the premature employment exit types unemployment, work disability, and early retirement are displayed, respectively. MetS increased the risk for unemployment (adjusted SHR: 1.15; 95% CI: 1.04–1.26) and work disability (adjusted SHR: 1.54; 95% CI: 1.30–1.83), but not for early retirement (Model 1 in tables 2–4). Similarly, poor SRH increased the risk for unemployment (adjusted SHR: 1.19; 95% CI: 1.04–1.36) and work disability (adjusted SHR: 5.40; 95% CI: 4.48–6.51), but not for early retirement (Model 2 in tables 2–4). After adding poor SRH to Model 1, the associations between MetS and unemployment (adjusted SHR: 1.14, 95% CI: 1.03–1.25) and work disability (adjusted SHR: 1.33, 95% CI: 1.11–1.58) were attenuated but remained statistically significant (Model 3 in tables 2 and 3).

**Table 2:** Prospective associations between MetS, SRH, and unemployment: competing risk regression analysis

	Model 1	Model 2	Model 3
	SHR (95% CI)	SHR (95% CI)	SHR (95% CI)
Health status			
MetS	1.15 (1.04, 1.26)		1.14 (1.03, 1.25)
Poor SRH		1.19 (1.04, 1.36)	1.17 (1.02, 1.34)
Chronic diseases			
Musculoskeletal	1.03 (0.84, 1.28)	1.02 (0.82, 1.26)	1.02 (0.82, 1.25)
Pulmonary	1.07 (0.96, 1.20)	1.07 (0.95, 1.20)	1.06 (0.95, 1.19)
Cancer	1.08 (0.92, 1.28)	1.08 (0.91, 1.28)	1.08 (0.91, 1.28)
Psychiatric	1.39 (1.26, 1.53)	1.37 (1.24, 1.51)	1.36 (1.23, 1.51)
Sociodemographic factors			
Age (years)	1.01 (1.00, 1.01)	1.01 (1.00, 1.02)	1.01 (1.00, 1.02)
Male sex	0.99 (0.90, 1.09)	1.00 (0.90, 1.10)	0.99 (0.89, 1.09)
Not married/partnered	1.57 (1.42, 1.73)	1.56 (1.41, 1.73)	1.56 (1.41, 1.73)
Occupation			
HSWC	Ref		
LSWC	1.27 (1.15, 1.40)	1.27 (1.15, 1.39)	1.27 (1.15, 1.39)
HSBC	0.95 (0.82, 1.09)	0.95 (0.82, 1.09)	0.95 (0.82, 1.09)
LSBC	1.17 (1.01, 1.34)	1.17 (1.01, 1.34)	1.16 (1.01, 1.34)
Education			
High	Ref		
Medium	1.20 (1.08, 1.34)	1.21 (1.09, 1.34)	1.20 (1.08, 1.34)
Low	1.56 (1.39, 1.76)	1.57 (1.40, 1.76)	1.56 (1.39, 1.75)
Other	1.28 (0.96, 1.69)	1.28 (0.97, 1.70)	1.28 (0.96, 1.69)
Working hours	1.00 (0.99, 1.00)	1.00 (0.99, 1.01)	1.00 (0.99, 1.01)

Note: Model 1 = MetS & covariates; Model 2 = SRH & covariates; Model 3 = MetS, SRH & covariates. SHR, subdistribution hazard ratio; 95% CI, 95% confidence interval; MetS, metabolic syndrome; SRH, self-rated health; HSWC, high-skilled white-collar; LSWC, low-skilled white-collar; HSBC, high-skilled blue-collar; LSBC, low-skilled blue-collar; Ref, reference group.

**Table 3:** Prospective associations between MetS, SRH, and work disability: competing risk regression analysis

	Model 1	Model 2	Model 3
	SHR (95% CI)	SHR (95% CI)	SHR (95% CI)
Health status			
MetS	1.54 (1.30, 1.83)		1.33 (1.11, 1.58)
Poor SRH		5.40 (4.48, 6.51)	5.24 (4.34, 6.32)
Chronic diseases			
Musculoskeletal	2.18 (1.63, 2.91)	1.63 (1.21, 2.18)	1.62 (1.20, 2.17)
Pulmonary	1.57 (1.29, 1.91)	1.28 (1.04, 1.57)	1.27 (1.03, 1.55)
Cancer	1.70 (1.29, 2.25)	1.57 (1.18, 2.08)	1.56 (1.18, 2.07)
Psychiatric	2.46 (2.08, 2.92)	1.81 (1.51, 2.17)	1.81 (1.51, 2.18)
Sociodemographic factors			
Age (years)	1.02 (1.01, 1.03)	1.03 (1.01, 1.04)	1.02 (1.01, 1.04)
Male sex	1.29 (1.02, 1.63)	1.23 (0.98, 1.54)	1.21 (0.96, 1.51)
Not married/partnered	1.52 (1.23, 1.87)	1.37 (1.11, 1.70)	1.37 (1.11, 1.69)
Occupation			
HSWC	Ref		
LSWC	1.01 (0.82, 1.25)	1.02 (0.83, 1.25)	1.02 (0.83, 1.25)
HSBC	1.88 (1.46, 2.43)	1.79 (1.38, 2.31)	1.80 (1.39, 2.33)
LSBC	1.73 (1.35, 2.23)	1.67 (1.30, 2.16)	1.66 (1.29, 2.14)
Education			
High	Ref		
Medium	1.22 (0.97, 1.53)	1.21 (0.97, 1.52)	1.20 (0.96, 1.50)
Low	1.67 (1.30, 2.13)	1.59 (1.24, 2.03)	1.55 (1.22, 1.98)
Other	1.21 (0.66, 2.21)	1.20 (0.65, 2.20)	1.17 (0.64, 2.16)
Working hours	0.96 (0.95, 0.97)	0.96 (0.95, 0.97)	0.96 (0.95, 0.97)

Note*:* Model 1 = MetS & covariates; Model 2 = SRH & covariates; Model 3 = MetS, SRH & covariates. SHR, subdistribution hazard ratio; 95% CI, 95% confidence interval; MetS, metabolic syndrome; SRH, self-rated health; HSWC, high-skilled white-collar; LSWC, low-skilled white-collar; HSBC, high-skilled blue-collar; LSBC, low-skilled blue-collar; Ref, reference group.

**Table 4:** Prospective associations between MetS, SRH, and early retirement: competing risk regression analysis

	Model 1	Model 2	Model 3
	SHR (95% CI)	SHR (95% CI)	SHR (95% CI)
Health status			
MetS	1.10 (0.94, 1.29)		1.10 (0.94, 1.29)
Poor SRH		1.00 (0.75, 1.32)	0.99 (0.75, 1.30)
Chronic diseases			
Musculoskeletal	1.13 (0.85, 1.49)	1.13 (0.85, 1.49)	1.13 (0.85, 1.49)
Pulmonary	0.77 (0.61, 0.96)	0.77 (0.61, 0.97)	0.77 (0.61, 0.97)
Cancer	1.12 (0.89, 1.41)	1.12 (0.89, 1.41)	1.12 (0.89, 1.41)
Psychiatric	1.18 (0.97, 1.44)	1.18 (0.97, 1.44)	1.18 (0.97, 1.44)
Sociodemographic factors			
Age (years)	1.38 (1.36, 1.39)	1.38 (1.36, 1.39)	1.38 (1.36, 1.39)
Male sex	1.06 (0.90, 1.24)	1.06 (0.91, 1.25)	1.06 (0.90, 1.24)
Not married/partnered	0.52 (0.40, 0.67)	0.52 (0.40, 0.67)	0.52 (0.40, 0.67)
Occupation			
HSWC	Ref		
LSWC	0.73 (0.62, 0.87)	0.73 (0.62, 0.87)	0.73 (0.62, 0.87)
HSBC	0.61 (0.47, 0.79)	0.61 (0.47, 0.79)	0.61 (0.47, 0.79)
LSBC	0.47 (0.35, 0.64)	0.48 (0.35, 0.65)	0.47 (0.35, 0.64)
Education			
High	Ref		
Medium	0.88 (0.74, 1.05)	0.89 (0.75, 1.05)	0.88 (0.74, 1.05)
Low	0.89 (0.75, 1.07)	0.90 (0.75, 1.07)	0.89 (0.75, 1.07)
Other	0.74 (0.44, 1.24)	0.74 (0.45, 1.24)	0.74 (0.44, 1.24)
Working hours	0.98 (0.98, 0.99)	0.98 (0.98, 0.99)	0.98 (0.98, 0.99)

Note*:* Model 1 = MetS & covariates; Model 2 = SRH & covariates; Model 3 = MetS, SRH & covariates. SHR, subdistribution hazard ratio; 95% CI, 95% confidence interval; MetS, metabolic syndrome; SRH, self-rated health; HSWC, high-skilled white-collar; LSWC, low-skilled white-collar; HSBC, high-skilled blue-collar; LSBC, low-skilled blue-collar; Ref, reference group.

### Sensitivity analyses

The main study findings hold when all working participants, i.e., also participants who worked 1 h or more but <12 h per week at baseline (*N* = 3841), were included in the analysis (Supplementary tables S2–S4). Further, when different codings of the risk factors MetS and SRH were used, comparable results were found (Supplementary tables S5–S10). Lastly, while all five MetS components significantly increased the risk for work disability, three MetS components increased the risk for unemployment (i.e., abdominal obesity, reduced HDL-cholesterol, and raised blood glucose) and only one MetS component (i.e., HDL-cholesterol) increased the risk for early retirement (Supplementary table S11).

## Discussion

Among 55 016 middle-aged and older Dutch workers, MetS was identified as an independent early-stage risk factor for premature employment exit while controlling for poor SRH, common chronic diseases related to labor market participation, and sociodemographic factors. More specifically, in the fully adjusted model, MetS remained a risk factor for work disability and unemployment during 4.3 years of follow-up.

The finding that MetS independently added to the risk for work disability and unemployment suggests that poor SRH only captures a ‘conscious’ part of the risk for premature employment exit. The other premature employment risk part associated with MetS is ‘hidden’, i.e., not fully reflected in SRH measures. Consequently, an assessment of poor SRH alone might lead to an underestimation of future labor market exit risks. Earlier research has shown the relevance of including externally assessed health indicators, like grip strength and BMI, additionally to SRH when examining labor market exit risks.^15^^,^^16^ Our findings add to the literature by showing that MetS, an externally assessed cardio-metabolic health indicator, is relevant to include as well.

The implications of this study for policy and practice are 3-fold. First, more awareness about MetS as a ‘hidden’ risk factor for premature employment exit is needed among workers, employers, and occupational health professionals. The growing global MetS epidemic^19^ and the baseline MetS prevalence found in this study (15.2%), which was more than twice as high as the prevalence of poor SRH (6.5%), underline the relevance of this cardio-metabolic health measure. Second, MetS prevention throughout the working life could contribute to longer working lives in good health. MetS development is closely linked to unhealthy behaviors. Individual motivation to change toward a healthier lifestyle—although difficult—has been identified as a major factor in reducing MetS components.^20^^,^^39^ Consequently, creating living and work environments that promote healthy behaviors might facilitate MetS prevention. Third, our findings suggest that it is valuable to include a MetS assessment in addition to SRH measures in health check-ups. Our sensitivity analyses showed that three of the individual MetS components increased the risk for unemployment while all five MetS components increased the risk for work disability. We acknowledge, though, that a full assessment of all MetS components, including blood testing, might not always be feasible in practice. Consequently, occupational physicians and general practitioners might start by measuring the MetS components abdominal obesity and hypertension. These two MetS components have been identified as main drivers for the increase in MetS prevalence with age in earlier research^40^ and were the most prevalent MetS components in this study. In case, a worker classifies for both, abdominal obesity and hypertension, further blood testing for a full MetS diagnosis may be valuable. More research is needed into other potentially ‘hidden’ risk factors for premature employment exit, such as biomarkers, which are not included in MetS. Further, the identification of driving MetS components for premature employment exit and the specific pathways and linking mechanisms between MetS and premature employment exit might help to inform preventive occupational health measures.

This study has some noteworthy strengths. The longitudinal study design including a clear temporal order of exposure and outcome measures from five waves of the Lifelines Cohort Study and Biobank limits the risk of reverse causation. Further, a unique combination of MetS and self-reported health measures was investigated to examine premature employment exit risks among a large sample of middle-aged and older workers. Lastly, the Lifelines population is broadly representative of the population of the North of the Netherlands, which facilitates the generalizability of results.^24^ A limitation concerns the limited follow-up time of 4.3 years during which employment states have only been assessed every 1.5 years. Longer follow-up studies linked to monthly register data on employment status would allow the examination of long-term effects of MetS and poor SRH on labor market participation and more detailed employment trajectories.

To conclude, MetS was identified as an independent ‘hidden’ cardio-metabolic risk factor for premature employment exit, i.e., the risk was not fully captured by poor SRH. This finding suggests that an assessment of SRH alone does not lead to an accurate estimation of future labor market exit risks. Additionally, it seems relevant to include externally assessed cardio-metabolic health indicators when investigating the risk for future work disability and unemployment. More awareness is needed among workers, employers, and occupational health practitioners about MetS as a ‘hidden’ risk factor for premature employment exit. Regular health check-ups including an assessment of MetS and MetS prevention might help to prolong healthy working lives.

## Supplementary Material

ckad219_Supplementary_Data

## Data Availability

Data are available on reasonable request. Data may be obtained from a third party and are not publicly available. Researchers can apply for the data and biomaterial through a proposal that is submitted to the Lifelines research office (research@lifelines.nl). Key points Poor self-rated health and chronic diseases only capture a part of premature employment exit risks. Metabolic syndrome (MetS) constitutes a ‘hidden’ risk factor for premature employment exit. Middle-aged and older workers with MetS are at risk for future work disability and unemployment. Increased MetS awareness, MetS prevention, and regular health check-ups might contribute to longer and healthier working lives.
