# Supplementary material for: Metabolic syndrome and poor self-rated health as risk factors for premature employment exit: a longitudinal study among 55 016 middle-aged and older workers from the Lifelines Cohort Study and Biobank
Source: Eur J Public Health. 2023 Dec 18;34(2):309–15. doi: 10.1093/eurpub/ckad219 (PMC10990532; doi:10.1093/eurpub/ckad219)
Supplement: ckad219_Supplementary_Data [file ckad219_supplementary_data.pdf]

Supplemental tables: Metabolic syndrome and poor self-rated health as risk factors for premature employment exit: A longitudinal study among 55,016 middle-aged and older workers from the Lifelines Cohort Study and Biobank

Supplemental table 1: Baseline (T0) characteristics of the total study sample and of participants who dropped-out or had information on employment status (ES) after T0

	**Total study sample** N=55,016	**Drop-out sample** N=4,587	**Missing ES sample** N=595
	% or mean (SD)	% or mean (SD)	% or mean (SD)
**Health status**			
MetS	15.2	16.8	17.1
MetS components			
Abdominal obesity	35.1	39.6	35.8
Hypertension	42.8	44.3	40.8
Raised triglycerides	18.9	19.6	22.0
Reduced HDL-Cholesterol	14.6	15.6	15.3
Raised blood glucose	12.3	12.3	13.1
Poor self-rated health	6.5	10.8	10.9
Chronic diseases			
Musculoskeletal	2.7	2.7	2.9
Pulmonary	10.4	12.5	11.1
Cancer	4.2	4.2	4.2
Psychiatric	11.3	13.5	10.4
**Sociodemographic factors**			
Age (years)	48.1 (5.7)	46.3 (4.9)	46.6 (4.9)
Male sex	45.8	49.7	57.3
Not married / partnered	10.0	11.9	11.3
Occupational group			
High skilled white-collar	50.0	43.4	46.9
Low skilled white-collar	31.0	32.1	26.2
High skilled blue-collar	10.7	13.0	13.0
Low skilled blue-collar	8.2	11.5	13.9
Educational level			
High	31.0	24.7	27.5
Medium	40.0	38.4	38.1
Low	27.4	35.1	30.9
Other	1.7	1.7	3.5
Weekly working hours	31.4 (8.7)	32.3 (8.7)	33.2 (8.4)

Abbreviations: SD, standard deviation; MetS, metabolic syndrome; HDL, high-density lipoprotein

Supplemental table 2: Prospective associations between MetS, SRH and unemployment: competing risk regression analysis among the total working study population (N=58,857)

	Model 1<br>SHR (95% CI)	Model 2<br>SHR (95% CI)	Model 3<br>SHR (95% CI)
**Health status**			
MetS	1.13 (1.03, 1.24)		1.12 (1.02, 1.23)
Poor SRH		1.20 (1.06, 1.36)	1.19 (1.05, 1.35)
Chronic diseases			
Musculoskeletal	1.00 (0.82, 1.21)	0.98 (0.80, 1.19)	0.98 (0.80, 1.19)
Pulmonary	1.06 (0.95, 1.18)	1.05 (0.94, 1.17)	1.04 (0.93, 1.16)
Cancer	1.06 (0.90, 1.24)	1.05 (0.90, 1.24)	1.05 (0.90, 1.24)
Psychiatric	1.34 (1.22, 1.47)	1.32 (1.20, 1.45)	1.32 (1.20, 1.45)
**Sociodemographic factors**			
Age (years)	1.01 (1.00, 1.01)	1.01 (1.00, 1.01)	1.01 (1.00, 1.01)
Male sex	1.03 (0.94, 1.13)	1.04 (0.94, 1.13)	1.03 (0.94, 1.13)
Not married / partnered	1.61 (1.47, 1.77)	1.61 (1.46, 1.77)	1.60 (1.46, 1.76)
Occupation			
HSWC	Ref		
LSWC	1.28 (1.17, 1.41)	1.28 (1.17, 1.41)	1.28 (1.17, 1.41)
HSBC	0.95 (0.83, 1.08)	0.94 (0.82, 1.08)	0.94 (0.82, 1.08)
LSBC	1.22 (1.07, 1.39)	1.22 (1.07, 1.39)	1.22 (1.07, 1.39)
Education			
High	Ref		
Medium	1.17 (1.06, 1.30)	1.18 (1.06, 1.30)	1.17 (1.06, 1.30)
Low	1.53 (1.37, 1.70)	1.53 (1.37, 1.71)	1.52 (1.36, 1.70)
Other	1.26 (0.97, 1.64)	1.26 (0.97, 1.64)	1.26 (0.96, 1.64)
Working hours	0.99 (0.99, 1.00)	0.99 (0.99, 1.00)	0.99 (0.99, 1.00)

Abbreviations: SHR, subdistribution hazard ratio; CI, confidence interval; MetS, metabolic syndrome; SRH, self-rated health; HSWC, high skilled white-collar; LSWC, low skilled white-collar; HSBC, high skilled blue-collar; LSBC, low skilled blue-collar; Ref, reference group

Note: Model 1 = MetS & covariates; Model 2 = SRH & covariates; Model 3 = MetS, SRH, & covariates

Supplemental table 3: Prospective associations between MetS, SRH and work disability: competing risk regression analysis among the total working study population (N=58,857)

	Model 1<br>SHR (95% CI)	Model 2<br>SHR (95% CI)	Model 3<br>SHR (95% CI)
**Health status**			
MetS	1.55 (1.33, 1.81)		1.32 (1.13, 1.55)
Poor SRH		5.31 (4.54, 6.20)	5.14 (4.40, 6.02)
Chronic diseases			
Musculoskeletal	2.06 (1.58, 2.67)	1.60 (1.24, 2.08)	1.61 (1.24, 2.09)
Pulmonary	1.66 (1.39, 1.98)	1.37 (1.15, 1.64)	1.36 (1.14, 1.63)
Cancer	1.68 (1.30, 2.16)	1.55 (1.20, 2.00)	1.55 (1.20, 2.00)
Psychiatric	2.46 (2.11, 2.86)	1.83 (1.56, 2.15)	1.83 (1.56, 2.15)
**Sociodemographic factors**			
Age (years)	1.01 (1.00, 1.02)	1.02 (1.01, 1.03)	1.02 (1.01, 1.03)
Male sex	1.43 (1.18, 1.74)	1.35 (1.12, 1.62)	1.32 (1.10, 1.59)
Not married / partnered	1.64 (1.36, 1.97)	1.50 (1.24, 1.81)	1.49 (1.24, 1.80)
Occupation			
HSWC	Ref		
LSWC	1.00 (0.83, 1.21)	1.02 (0.84, 1.22)	1.02 (0.84, 1.23)
HSBC	1.80 (1.43, 2.27)	1.75 (1.38, 2.21)	1.76 (1.39, 2.22)
LSBC	1.40 (1.11, 1.76)	1.39 (1.11, 1.75)	1.38 (1.10, 1.73)
Education			
High	Ref		
Medium	1.30 (1.05, 1.60)	1.28 (1.04, 1.58)	1.27 (1.03, 1.56)
Low	1.71 (1.36, 2.15)	1.62 (1.29, 2.03)	1.58 (1.26, 1.98)
Other	1.31 (0.78, 2.21)	1.30 (0.77, 2.20)	1.27 (0.75, 2.15)
Working hours	0.96 (0.95, 0.96)	0.96 (0.95, 0.97)	0.96 (0.95, 0.97)

Abbreviations: SHR, subdistribution hazard ratio; CI, confidence interval; MetS, metabolic syndrome; SRH, self-rated health; HSWC, high skilled white-collar; LSWC, low skilled white-collar; HSBC, high skilled blue-collar; LSBC, low skilled blue-collar; Ref, reference group

Note: Model 1 = MetS & covariates; Model 2 = SRH & covariates; Model 3 = MetS, SRH, & covariates

Supplemental table 4: Prospective associations between MetS, SRH and early retirement: competing risk regression analysis among the total working study population (N=58,857)

	Model 1<br>SHR (95% CI)	Model 2<br>SHR (95% CI)	Model 3<br>SHR (95% CI)
**Health status**			
MetS	1.05 (0.90, 1.21)		1.05 (0.90, 1.21)
Poor SRH		0.99 (0.76, 1.30)	0.99 (0.76, 1.29)
Chronic diseases			
Musculoskeletal	1.05 (0.81, 1.37)	1.05 (0.81, 1.37)	1.06 (0.81, 1.38)
Pulmonary	0.77 (0.62, 0.96)	0.78 (0.63, 0.96)	0.78 (0.62, 0.96)
Cancer	1.12 (0.90, 1.39)	1.12 (0.90, 1.39)	1.12 (0.90, 1.39)
Psychiatric	1.21 (1.00, 1.46)	1.21 (1.00, 1.46)	1.21 (1.00, 1.46)
**Sociodemographic factors**			
Age (years)	1.37 (1.36, 1.38)	1.37 (1.36, 1.38)	1.37 (1.36, 1.38)
Male sex	1.00 (0.86, 1.16)	1.00 (0.87, 1.16)	1.00 (0.86, 1.16)
Not married / partnered	0.52 (0.41, 0.67)	0.52 (0.41, 0.67)	0.52 (0.41, 0.67)
Occupation			
HSWC	Ref		
LSWC	0.72 (0.61, 0.85)	0.72 (0.62, 0.85)	0.72 (0.61, 0.85)
HSBC	0.60 (0.47, 0.76)	0.60 (0.47, 0.76)	0.60 (0.47, 0.76)
LSBC	0.49 (0.38, 0.64)	0.49 (0.38, 0.64)	0.49 (0.38, 0.64)
Education			
High	Ref		
Medium	0.92 (0.78, 1.08)	0.92 (0.79, 1.09)	0.92 (0.78, 1.08)
Low	0.90 (0.76, 1.07)	0.90 (0.76, 1.07)	0.90 (0.76, 1.07)
Other	0.86 (0.55, 1.36)	0.87 (0.55, 1.36)	0.86 (0.55, 1.36)
Working hours	1.00 (0.99, 1.00)	1.00 (0.99, 1.00)	1.00 (0.99, 1.00)

Abbreviations: SHR, subdistribution hazard ratio; CI, confidence interval; MetS, metabolic syndrome; SRH, self-rated health; HSWC, high skilled white-collar; LSWC, low skilled white-collar; HSBC, high skilled blue-collar; LSBC, low skilled blue-collar; Ref, reference group

Note: Model 1 = MetS & covariates; Model 2 = SRH & covariates; Model 3 = MetS, SRH, & covariates

Supplemental table 5: Prospective associations between number of MetS components, SRH and unemployment: competing risk regression analysis

	Model 1<br>SHR (95% CI)	Model 2<br>SHR (95% CI)
**Health status**		
Number of MetS components		
0	0.81 (0.71, 0.92)	0.81 (0.72, 0.92)
1	0.88 (0.78, 1.00)	0.89 (0.79, 1.01)
2	0.91 (0.80, 1.04)	0.91 (0.80, 1.04)
3	Ref	
4	0.95 (0.78, 1.15)	0.94 (0.78, 1.14)
5	1.06 (0.78, 1.44)	1.06 (0.78, 1.43)
Poor SRH		1.16 (1.01, 1.33)
Chronic diseases		
Musculoskeletal	1.03 (0.84, 1.27)	1.01 (0.82, 1.25)
Pulmonary	1.07 (0.95, 1.20)	1.06 (0.94, 1.19)
Cancer	1.08 (0.92, 1.28)	1.08 (0.91, 1.28)
Psychiatric	1.39 (1.26, 1.53)	1.36 (1.23, 1.51)
**Sociodemographic factors**		
Age (years)	1.01 (1.00, 1.01)	1.01 (1.00, 1.01)
Male sex	0.98 (0.89, 1.09)	0.98 (0.89, 1.08)
Not married / partnered	1.57 (1.42, 1.73)	1.56 (1.41, 1.73)
Occupation		
HSWC	Ref	
LSWC	1.26 (1.15, 1.39)	1.26 (1.15, 1.39)
HSBC	0.95 (0.82, 1.09)	0.95 (0.82, 1.09)
LSBC	1.16 (1.01, 1.34)	1.16 (1.01, 1.34)
Education		
High	Ref	
Medium	1.19 (1.08, 1.33)	1.19 (1.08, 1.33)
Low	1.55 (1.38, 1.74)	1.54 (1.37, 1.73)
Other	1.26 (0.95, 1.68)	1.26 (0.95, 1.68)
Working hours	1.00 (0.99, 1.00)	1.00 (0.99, 1.00)

Abbreviations: SHR, subdistribution hazard ratio; CI, confidence interval; MetS, metabolic syndrome; SRH, self-rated health; HSWC, high skilled white-collar; LSWC, low skilled white-collar; HSBC, high skilled blue-collar; LSBC, low skilled blue-collar; Ref, reference group

Note: Model 1 = Number of MetS components & covariates; Model 2 = Number of MetS components, SRH, & covariates

Supplemental table 6: Prospective associations between number of MetS components, SRH and work disability: competing risk regression analysis

	Model 1<br>SHR (95% CI)	Model 2<br>SHR (95% CI)
**Health status**		
Number of MetS components		
0	0.67 (0.51, 0.87)	0.78 (0.59, 1.02)
1	0.87 (0.68, 1.12)	0.98 (0.76, 1.27)
2	1.01 (0.77, 1.31)	1.06 (0.81, 1.38)
3	Ref	
4	1.75 (1.27, 2.41)	1.59 (1.15, 2.21)
5	1.90 (1.17, 3.08)	1.71 (1.06, 2.76)
Poor SRH		5.11 (4.23, 6.17)
Chronic diseases		
Musculoskeletal	2.13 (1.59, 2.85)	1.60 (1.19, 2.16)
Pulmonary	1.55 (1.27, 1.89)	1.26 (1.02, 1.54)
Cancer	1.69 (1.28, 2.23)	1.56 (1.17, 2.07)
Psychiatric	2.44 (2.05, 2.89)	1.80 (1.50, 2.16)
**Sociodemographic factors**		
Age (years)	1.02 (1.00, 1.03)	1.02 (1.01, 1.03)
Male sex	1.26 (1.00, 1.59)	1.19 (0.95, 1.49)
Not married / partnered	1.51 (1.22, 1.85)	1.37 (1.11, 1.69)
Occupation		
HSWC	Ref	
LSWC	1.01 (0.82, 1.24)	1.01 (0.82, 1.25)
HSBC	1.89 (1.47, 2.45)	1.81 (1.40, 2.34)
LSBC	1.70 (1.32, 2.19)	1.64 (1.27, 2.11)
Education		
High	Ref	
Medium	1.18 (0.94, 1.49)	1.18 (0.94, 1.48)
Low	1.60 (1.25, 2.05)	1.51 (1.18, 1.93)
Other	1.16 (0.64, 2.13)	1.15 (0.63, 2.12)
Working hours	0.96 (0.95, 0.97)	0.96 (0.95, 0.97)

Abbreviations: SHR, subdistribution hazard ratio; CI, confidence interval; MetS, metabolic syndrome; SRH, self-rated health; HSWC, high skilled white-collar; LSWC, low skilled white-collar; HSBC, high skilled blue-collar; LSBC, low skilled blue-collar; Ref, reference group

Note: Model 1 = Number of MetS components & covariates; Model 2 = Number of MetS components, SRH, & covariates

Supplemental table 7: Prospective associations between number of MetS components, SRH and early retirement: competing risk regression analysis

	Model 1 SHR (95% CI)	Model 2 SHR (95% CI)
**Health status**		
Number of MetS components		
0	0.96 (0.77, 1.20)	0.96 (0.77, 1.20)
1	0.92 (0.74, 1.14)	0.92 (0.74, 1.14)
2	1.05 (0.84, 1.31)	1.05 (0.84, 1.31)
3	Ref	
4	1.14 (0.84, 1.56)	1.14 (0.84, 1.56)
5	1.33 (0.85, 2.07)	1.33 (0.85, 2.07)
Poor SRH		0.98 (0.74, 1.29)
Chronic diseases		
Musculoskeletal	1.12 (0.85, 1.49)	1.13 (0.85, 1.49)
Pulmonary	0.77 (0.61, 0.96)	0.77 (0.61, 0.97)
Cancer	1.12 (0.89, 1.40)	1.12 (0.89, 1.40)
Psychiatric	1.18 (0.97, 1.44)	1.18 (0.97, 1.44)
**Sociodemographic factors**		
Age (years)	1.37 (1.36, 1.39)	1.37 (1.36, 1.39)
Male sex	1.06 (0.90, 1.24)	1.06 (0.90, 1.24)
Not married / partnered	0.51 (0.40, 0.67)	0.51 (0.40, 0.67)
Occupation		
HSWC	Ref	
LSWC	0.73 (0.62, 0.87)	0.73 (0.62, 0.87)
HSBC	0.62 (0.48, 0.80)	0.62 (0.48, 0.80)
LSBC	0.47 (0.35, 0.64)	0.47 (0.35, 0.64)
Education		
High	Ref	
Medium	0.88 (0.74, 1.04)	0.88 (0.74, 1.04)
Low	0.89 (0.74, 1.06)	0.89 (0.74, 1.06)
Other	0.74 (0.44, 1.23)	0.74 (0.44, 1.23)
Working hours	0.98 (0.98, 0.99)	0.98 (0.98, 0.99)

Abbreviations: SHR, subdistribution hazard ratio; CI, confidence interval; MetS, metabolic syndrome; SRH, self-rated health; HSWC, high skilled white-collar; LSWC, low skilled white-collar; HSBC, high skilled blue-collar; LSBC, low skilled blue-collar; Ref, reference group

Note: Model 1 = Number of MetS components & covariates; Model 2 = Number of MetS components, SRH, & covariates

Supplemental table 8: Prospective associations between MetS, SRH (continuous) and unemployment: competing risk regression analysis

	Model 1<br>SHR (95% CI)	Model 2<br>SHR (95% CI)
**Health status**		
MetS		1.12 (1.02, 1.23)
SRH	1.10 (1.05, 1.16)	1.10 (1.04, 1.15)
Chronic diseases		
Musculoskeletal	1.00 (0.81, 1.24)	1.00 (0.81, 1.24)
Pulmonary	1.05 (0.94, 1.18)	1.05 (0.94, 1.18)
Cancer	1.07 (0.91, 1.27)	1.08 (0.91, 1.27)
Psychiatric	1.35 (1.22, 1.49)	1.35 (1.22, 1.49)
**Sociodemographic factors**		
Age (years)	1.01 (1.00, 1.02)	1.01 (1.00, 1.02)
Male sex	1.00 (0.90, 1.10)	0.99 (0.89, 1.09)
Not married / partnered	1.56 (1.41, 1.72)	1.55 (1.40 (1.72)
Occupation		
HSWC	Ref	
LSWC	1.26 (1.15, 1.39)	1.26 (1.15, 1.39)
HSBC	0.94 (0.82, 1.08)	0.94 (0.82, 1.08)
LSBC	1.16 (1.01, 1.34)	1.16 (1.00, 1.33)
Education		
High	Ref	
Medium	1.20 (1.08, 1.33)	1.19 (1.07, 1.32)
Low	1.55 (1.38, 1.74)	1.54 (1.37, 1.72)
Other	1.27 (0.96, 1.68)	1.26 (0.95, 1.67)
Working hours	1.00 (0.99, 1.01)	1.00 (0.99, 1.01)

Abbreviations: SHR, subdistribution hazard ratio; CI, confidence interval; MetS, metabolic syndrome; SRH, self-rated health; HSWC, high skilled white-collar; LSWC, low skilled white-collar; HSBC, high skilled blue-collar; LSBC, low skilled blue-collar; Ref, reference group
Note: Model 1 = SRH & covariates; Model 2 = MetS, SRH, & covariates

Supplemental table 9: Prospective associations between MetS, SRH (continuous) and work disability: competing risk regression analysis

	Model 1<br>SHR (95% CI)	Model 2<br>SHR (95% CI)
**Health status**		
MetS		1.24 (1.04, 1.48)
SRH	3.00 (2.58, 3.49)	2.95 (2.53, 3.44)
Chronic diseases		
Musculoskeletal	1.51 (1.11, 2.04)	1.50 (1.10, 2.04)
Pulmonary	1.23 (1.01, 1.51)	1.22 (1.00, 1.50)
Cancer	1.55 (1.17, 2.06)	1.55 (1.17, 2.05)
Psychiatric	1.76 (1.47, 2.10)	1.76 (1.47, 2.11)
**Sociodemographic factors**		
Age (years)	1.03 (1.01, 1.04)	1.02 (1.01, 1.04)
Male sex	1.21 (0.97, 1.52)	1.19 (0.95, 1.50)
Not married / partnered	1.33 (1.08, 1.65)	1.34 (1.08, 1.65)
Occupation		
HSWC	Ref	
LSWC	1.00 (0.81, 1.23)	1.00 (0.81, 1.23)
HSBC	1.70 (1.31, 2.20)	1.71 (1.32, 2.22)
LSBC	1.60 (1.24, 2.06)	1.60 (1.24, 2.06)
Education		
High	Ref	
Medium	1.14 (0.91, 1.43)	1.13 (0.90, 1.42)
Low	1.45 (1.13, 1.85)	1.43 (1.11, 1.82)
Other	1.12 (0.61, 2.05)	1.11 (0.60, 2.02)
Working hours	0.96 (0.95, 0.98)	0.96 (0.95, 0.98)

Abbreviations: SHR, subdistribution hazard ratio; CI, confidence interval; MetS, metabolic syndrome; SRH, self-rated health; HSWC, high skilled white-collar; LSWC, low skilled white-collar; HSBC, high skilled blue-collar; LSBC, low skilled blue-collar; Ref, reference group

Note: Model 1 = SRH & covariates; Model 2 = MetS, SRH, & covariates

Supplemental table 10: Prospective associations between MetS, SRH (continuous) and early retirement: competing risk regression analysis

	Model 1<br>SHR (95% CI)	Model 2<br>SHR (95% CI)
**Health status**		
MetS		1.09 (0.94, 1.28)
SRH	1.05 (0.97, 1.14)	1.05 (0.96, 1.14)
Chronic diseases		
Musculoskeletal	1.11 (0.84, 1.47)	1.11 (0.84, 1.47)
Pulmonary	0.76 (0.60, 0.96)	0.76 (0.60, 0.95)
Cancer	1.11 (0.89, 1.40)	1.12 (0.89, 1.40)
Psychiatric	1.16 (0.95, 1.42)	1.17 (0.96, 1.42)
**Sociodemographic factors**		
Age (years)	1.38 (1.36, 1.39)	1.38 (1.36, 1.39)
Male sex	1.06 (0.91, 1.24)	1.06 (0.90, 1.24)
Not married / partnered	0.52 (0.40, 0.67)	0.52 (0.40, 0.67)
Occupation		
HSWC	Ref	
LSWC	0.73 (0.62, 0.87)	0.73 (0.62, 0.87)
HSBC	0.61 (0.47, 0.79)	0.61 (0.47, 0.79)
LSBC	0.47 (0.35, 0.64)	0.47 (0.35, 0.64)
Education		
High	Ref	
Medium	0.88 (0.74, 1.04)	0.88 (0.74, 1.04)
Low	0.89 (0.75, 1.07)	0.89 (0.74, 1.06)
Other	0.74 (0.44, 1.23)	0.74 (0.44, 1.23)
Working hours	0.98 (0.98, 0.99)	0.98 (0.98, 0.99)

Abbreviations: SHR, subdistribution hazard ratio; CI, confidence interval; MetS, metabolic syndrome; SRH, self-rated health; HSWC, high skilled white-collar; LSWC, low skilled white-collar; HSBC, high skilled blue-collar; LSBC, low skilled blue-collar; Ref, reference group
Note: Model 1 = SRH & covariates; Model 2 = MetS, SRH, & covariates

Supplemental table 11: Prospective associations between individual MetS components and premature employment exit: competing risk regression analysis

	Unemployment SHR (95% CI)	Work disability SHR (95% CI)	Early retirement SHR (95% CI)
**MetS components**			
Abdominal obesity	1.09 (1.01, 1.17)	1.32 (1.13, 1.53)	1.04 (0.91, 1.19)
Hypertension	1.08 (1.00, 1.16)	1.17 (1.00, 1.36)	1.02 (0.90, 1.16)
Raised triglycerides	1.06 (0.96, 1.16)	1.26 (1.06, 1.50)	1.10 (0.95, 1.28)
Reduced HDL-Cholesterol	1.11 (1.00, 1.22)	1.40 (1.17, 1.67)	1.24 (1.05, 1.47)
Raised blood glucose	1.15 (1.04, 1.28)	1.25 (1.02, 1.52)	1.09 (0.93, 1.29)

Abbreviations: MetS, metabolic syndrome; SHR, subdistribution hazard ratio; CI, confidence interval
Note: MetS components were individually investigated in separate analyses; all models were adjusted for self-rated health, chronic diseases, age, sex, partner status, occupational group, education, and working hours
